# Consumers or Citizens? Whose Voice Will Healthwatch Represent and Will It Matter? Comment on "Challenges Facing Healthwatch, a New Consumer Champion in England"

**DOI:** 10.15171/ijhpm.2016.84

**Published:** 2016-06-22

**Authors:** Brad Wright

**Affiliations:** Department of Health Management and Policy, College of Public Health, University of Iowa, Iowa City, IA, USA.

**Keywords:** Patient and Public Involvement (PPI), Consumer Involvement, Governance, Representation, Healthwatch, England

## Abstract

Efforts to achieve effective and meaningful patient and public involvement (PPI) in healthcare have existed for nearly a century, albeit with limited success. This brief commentary discusses a recent paper by Carter and Martin exploring the *"Challenges Facing Healthwatch, a New Consumer Champion in England,"* and places these challenges in the context of the broader struggle to give a voice to healthcare consumers and citizens. With an overview of what can go right and—perhaps more importantly—what can go wrong, the question remains: will Healthwatch—and other PPI efforts in healthcare—represent the voice of consumers or citizens and will it matter?

From the regional health authorities of Canada to the district health boards of New Zealand and the foundation trusts of England to the health systems agencies of the United States, examples of democratic governments attempting to implement meaningful patient and public involvement (PPI) in healthcare abound.^1-7^ However, identifying successful efforts is more challenging, owing to the lack of a clear definition of PPI and conflicting rationales for its existence.^8^ While patients have occasionally proven able to contribute to healthcare planning and development,^9^ failures to implement effective consumer governance in healthcare can be found in Canada,^1^ the United Kingdom,^3^ and the United States.^2,4-6,10^ Yet the dream of achieving effective and meaningful PPI in healthcare—elusive as it seems to be—is still alive and well.^11^ These efforts have taken on yet another form in England, as Carter and Martin write in “*Challenges Facing Healthwatch, a New Consumer Champion in England*.”^12^

Formally established by the Health & Social Care Act 2012, Healthwatch proclaims its mission to be “the consumer champion for health and social care.” Healthwatch consists of both local bodies (Local Healthwatch) and a national body (Healthwatch England). The Local Healthwatch are given “a seat on the statutory health and well-being boards…[enabling] people to share their views and concerns about their local health and social care services…[alerting] Healthwatch England…to concerns about specific care providers, health or social care matters, [and providing] people with information about their choices and what to do when things go wrong…[including] information about local health and care services and how to access them.”^13^ Given England’s history of a string of PPI efforts over the last 40 years including Community Health Councils, PPI Forums, and Local Involvement Networks (LINks),^12^ the most pressing question may not be “Will Healthwatch succeed where others have failed?” but rather “Will Healthwatch represent the voice of healthcare consumers or citizens?” and most importantly, “Will it matter?”

PPI in healthcare hinges on the idea of representation, a concept which Pitkin delineated to include *formal*, *descriptive*, and *substantive* dimensions, which refer respectively to how representatives are chosen, the extent to which representatives resemble constituents, and the extent to which representatives accurately seek the interests of constituents.^14^ More plainly, successful PPI must be intentional about who is to participate, how they are selected, what role they are to play, and what ends they are to pursue.^15^ All too often, these questions are not thoroughly answered, and this appears to be the case with Healthwatch.

Although Healthwatch has formal authority by virtue of the fact that they are granted a seat on local health and well-being boards, the mechanism of formal representation is less clear. Typically, representatives are authorized and held accountable by those they represent through mechanisms of formal representation such as elections. However, Healthwatch jurisdictions are not necessarily defined according to traditional geopolitical boundaries (eg, counties). The end result is that the local community may be ill defined and represented by a variety of decision-makers and stakeholders at different levels, which may overlap to differing degrees, making it much more difficult for local citizens and/or consumers to have an effective voice.^12^

Moreover, it seems that the local people involved in Healthwatch are volunteers. Reliance on a voluntary model is concerning, as prior PPI failures can be traced in part to challenges getting an adequate number of consumers to participate.^16^ Furthermore, despite statutory requirements that local Healthwatch must be “inclusive and reflect the diversity of the community it serves,”^13^ costs inherent in participation are likely to pose a barrier to those who may be lower income or otherwise disadvantaged. Consequently, these individuals may find that their voice is excluded from the discussion altogether. Even in the midst of inclusion, exclusion can be prevalent. As status generalization theory suggests, small groups recreate the power dynamics found in society at large, which tends to amplify the voices of those with higher social standing and drown out the voices of the meek.^17-19^ Thus, efforts at exclusion may be just as important as efforts at inclusion. Political scientist, Suzanne Dovi, stresses that “Not only do some voices need to be brought in, some voices need to be muted.”^20^ Notably, there is evidence that further formalizing representation through the introduction of elections is unlikely to remedy the limitations inherent in this voluntary model of PPI.^7,21^

Next, there is the question of why certain individuals are selected for participation. For example, service users may conceivably participate as individual consumers of services, as advocates for a group of consumers, or as citizens representing the community.^22^ As Carter and Martin suggest, there are conflicting messages as to whether Healthwatch is to be a “consumer champion” or a mechanism to “strengthen the collective voice of local people.”^12^ Further complicating matters, consumers are not a homogeneous group and certain types of consumers are more likely to engage in PPI activities than others.^23^

There is also an inherent paradox between the intention and the implementation of PPI manifested in seeking the involvement of ‘ordinary’ patients, consumer advocates, or public citizens who possess the extraordinary ability to be effective representatives.^24^ John Gaventa reminds us that “mandates for participation from ‘above’ must be linked with pre-existing capacities for participation from ‘below’”^25^ and many have argued that lay persons simply lack the requisite skills to be effective participants in governance and other decision-making settings.^26-29^ Consequently, there is an ongoing debate about whether such ‘ordinary’ individuals can play a meaningful role in healthcare governance and decision-making without first being co-opted by established interests.^30,31^

Finally, there is the question of what ultimate influence we expect participants to have on measurable outcomes. The law stipulates that local Healthwatch are tasked with obtaining the “views of local people regarding health and care services” and relaying that information to decision-makers.^12^ However, this is left open to interpretation. As Carter and Martin discuss, this could be consistent with promoting voice among the local citizenry or promoting choice among consumers.^12^ There is likely to be variation in interpretation across local Healthwatch, which poses a challenge. Without knowing expressly what Healthwatch is intended to do, it becomes difficult to know whether it is achieving the intended outcome. As Carter and Martin summarize it: “If action does not result, apparent avenues for voice might turn out to be culs-de-sac or a ‘dialogue of the deaf.’”^12^

David Brindle writes in *The Guardian* that the Staffordshire hospitals scandal “reminds us of the critical importance of PPI in the care system – and of what can happen when the voices of patients and family carers are not heard.”^32^ Indeed, Healthwatch makes clear that in all of the recent scandals (eg, Mid-Staffordshire, Morecambe Bay, and Winterbourne View), “local people were raising concerns and worries long before they were properly listened to.”^33^ Clearly there is an ongoing desire to give patients and citizens a voice in healthcare governance, but merely replacing one program with another when dissatisfaction reaches a tipping point is unlikely to bring England—or any other nation—closer to realizing that desire.

To be certain, Healthwatch—like other PPI efforts—faces a host of challenges, including a struggle to demonstrate legitimacy, limited financial resources, conflicts of interest vis-à-vis the established interests of the healthcare system, and the risk of being co-opted by those very same interests.^12^ However, until policy-makers intentionally design PPI efforts that are clear about who participates, how they are selected, why they have been selected (ie, as consumers or citizens), what activities are within their purview, and how they will be held accountable by those they are intended to represent, it is doubtful that the voice of consumers or citizens will ever be brought to bear upon healthcare in a truly meaningful way.

## Ethical issues

Not applicable.

## Competing interests

Author declares that he has no competing interests.

## Author’s contribution

BW is the single author of the paper.
